# A *q*-Extension of Sigmoid Functions and the Application for Enhancement of Ultrasound Images

**DOI:** 10.3390/e21040430

**Published:** 2019-04-23

**Authors:** Paulo Sergio Rodrigues, Guilherme Wachs-Lopes, Ricardo Morello Santos, Eduardo Coltri, Gilson Antonio Giraldi

**Affiliations:** 1 Computer Science Department, Centro Universitário FEI, São Bernardo do Campo 09850-901, SP, Brazil; 2 National Laboratory for Scientific Computing, Petrópolis 25651-075, RJ, Brazil

**Keywords:** contrast enhancement, sigmoid, Tsallis statistics, *q*-exponential, *q*-sigmoid, *q*-Gaussian, ultra-sound images

## Abstract

This paper proposes the *q*-sigmoid functions, which are variations of the sigmoid expressions and an analysis of their application to the process of enhancing regions of interest in digital images. These new functions are based on the non-extensive Tsallis statistics, arising in the field of statistical mechanics through the use of *q*-exponential functions. The potential of *q*-sigmoids for image processing is demonstrated in tasks of region enhancement in ultrasound images which are highly affected by speckle noise. Before demonstrating the results in real images, we study the asymptotic behavior of these functions and the effect of the obtained expressions when processing synthetic images. In both experiments, the *q*-sigmoids overcame the original sigmoid functions, as well as two other well-known methods for the enhancement of regions of interest: slicing and histogram equalization. These results show that *q*-sigmoids can be used as a preprocessing step in pipelines including segmentation as demonstrated for the Otsu algorithm and deep learning approaches for further feature extractions and analyses.

## 1. Introduction

The use of sigmoid functions has been one of the most prominent strategies in the field of digital image processing, applied not only to highlight characteristics of scenes, objects, or regions of interest but also to segment different image modalities [1,2]. In this paper, we focus on sigmoid functions applied to the enhancement of regions of interest or to the enhancement of global contrast. In this way, the work of Reference [3] proposes an image enhancement method using a modified sigmoid function, where a 3×3 kernel was used as a low-pass convolution filter in the *Y* component of the YCbCr color system. In the same line, in addition to using the sigmoid function as a filter, Reference [4] proposed the improvement of the support vector regression (SVR) method using a sigmoid kernel for high-resolution imaging without the need for training datasets. In this approach, the best models were chosen using the Bayesian decision theory.

For segmentation, the work of Reference [5] presented a neural network with a nucleus based on a sigmoid function in order to segment cysts in liver tissues. Also, in Reference [6], methodology called group method of data handling (GMHD) for malignant tumors detection in computed tomography is applied. However, considering the low contrast in some medical CT scans, the segmentation of organs, tumors, or other types of components becomes difficult tasks and wrong results can compromise diagnoses.

Such a problem motivates the study that can be found in Reference [7], which proposed a contrast enhancement algorithm and compares its performance with counterpart methods for several modalities of images. On the other hand, the work in Reference [8] presents a sigmoid model based on a cross-correlation algorithm to solve estimation errors in ultrasound images. More recently, saliency models and convolutional neural networks (CNNs) have been used for automatic tumor detection and breast segmentation and in ultrasound images [9,10].

Beyond the medical images field, another application of sigmoid functions is in the area of image fusion for the global enhancement of focus or contrast. Thus, Reference [11] presented a method of merging several partially focused images into different regions for generating a globally focused image using a sigmoid function. Moreover, in Reference [12], it is proposed a fusion algorithm of multifocal imaging based on a spectral comparison. In this algorithm, a sigmoid function was also applied in the fusion process. Also in the same line, the work by Reference [13] presented an image fusion method based on the segmentation of regions with the use of a sigmoid function applied to the method named adaptive multi-strategy fusion rule (AMFR).

In addition to segmentation and fusion of images, another important application of sigmoid functions is for the improvement of stereoscopic images. In this way, Reference [14] presented a visual comfort enhancement approach in 3-D stereoscopic images using nonlinear disparity mapping adaptable to fiducial features.

However, the efficiency of sigmoid functions in the mentioned applications depends mainly on the parameterization involved, which in turn leads the curve fitting over the regions of interest. On the other hand, the flexibility of sigmoid functions, based on traditional exponential and Gaussian functions, is restricted to the standard deviation and mean, imposing limitations due to reduced degrees of freedom. However, in the mid-1980s, the Tsallis entropy approach allowed more general functions to be proposed in the field of non-extensive statistical mechanics, also called *q*-statistics, which proved to be a generalization of the traditional statistics [15,16,17]. Since its initial proposal, this theory has been accepted in many areas of applications [18]. In the medical imaging field, in particular, it has been successfully used for feature extraction [19] but mainly for image segmentation [20,21,22,23]. Also, it allowed the emergence of the *q*-exponential functions, of which the parameter $q\in I\;R^{+}$, called the non-extensive parameter, can be used for fine tuning the image information.

Thus, in this paper, we introduce the *q*-exponential as a kernel of sigmoid expression, generating the *q*-sigmoid functions. Then, we demonstrate the versatility of *q*-sigmoids in contrast enhancement. The *q*-sigmoids are applied in the core of the image processing algorithm, and the obtained results are compared with the original sigmoid functions, as well as two other well-known methods for image enhancement: slicing and histogram equalization. We have shown that their superior performance over traditional methods is achieved by adapting the profile of these new functions to the image intensity distributions involved. Moreover, we demonstrate the advantages of *q*-sigmoid as a preprocessing step of input images in segmentation pipelines that include Otsu thresholding or CNN architectures [24,25]. The U-Net is a known deep network in this field, outperforming prior best methods [10].

The paper is organized as follows. Section 2 focuses on the *q*-statistics approach and *q*-exponential functions and summarizes some of their basic properties. The *q*-sigmoids families are proposed in Section 3, and important properties of such functions for image enhancement are proved in Section 4. The computational results are presented in Section 5. Next, Section 6 discusses some important points that have emerged from this work, the relationship with related topics, and further improvements regarding automatic parametrization. Finally, in Section 7, we conclude the paper, summarizing its main contributions and describing possible future work.

## 2. The q-Exponential Distribution

In the last decades, Tsallis [16] has proposed the following generalized non-extensive entropic form:

(1) $$S_{q}=k\frac{1-\sum_{i=1}^{W}p_{i}^{q}}{q-1},$$

where *k* is a positive constant, $0\leq p_{i}\leq 1$ is a probability distribution, *W* is the total number of states of the system (grayscale intensities, in the case of digital imaging), and $q\in I\;R^{+}$ is called the entropic index, or *q*-index also. This expression recovers the Shannon entropy in the limit q→1. The Tsallis entropy, computed by Equation (1), offers a new formalism in which the real parameter *q* quantifies the level of nonextensivity of a physical systems [16]. In particular, a general principle of maximum entropy (PME) has been considered to find out the distribution $p_{i}$ to describe such systems. In this PME, the goal is to find the maximum of $S_{q}$ subjected to

(2) $$\sum_{i=1}^{W}p_{i}=1,$$

and

(3) $$\frac{\sum_{i=1}^{W}e_{i}p_{i}^{q}}{\sum_{i=1}^{W}p_{i}^{q}}=U_{q},$$

where $U_{q}$ is a known application dependent value and $e_{i}$ represents the possible states of the system (in image processing, the grayscale intensities). Equation (2) is just a necessary condition for $p_{i}$ to be probability, and Equation (3) is a generalized expectation value of the $e_{i}$ (if q=1, we get the usual mean value). The proposed PME can be solved using Lagrange multipliers, and the solution has the form [15,16]

(4) $$p_{j}=\frac{{\left[1-\left(1-q\right)\tilde{\beta }e_{j}\right]}^{\frac{1}{1-q}}}{{\tilde{Z}}_{q}},$$

where $\tilde{\beta }$ and ${\tilde{Z}}_{q}$ are defined by Equations (5) and (6):

(5) $$\tilde{\beta }=\frac{\beta }{\sum_{j=1}^{W}p_{j}^{q}+\left(1-q\right)\beta U_{q}},$$

(6) $${\tilde{Z}}_{q}=\sum_{j=1}^{W}{\left[1-\left(1-q\right)\tilde{\beta }e_{j}\right]}^{\frac{1}{1-q}},$$

with β being the Lagrange multiplier associated with the constraint given by Equation (3), and if q<1, then $p_{i}=0$ whenever $1-\left(1-q\right)\tilde{\beta }e_{j}<0$ (cutoff condition). Then, Equations (1) and (4) inspire the definition of the *q*-exponential function [26]:

(7) $$\begin{array}{c} \exp_{q}\left(x\right)=\left\{\begin{array}{cc} {\left[1+\left(1-q\right)x\right]}^{\frac{1}{1-q}}, & \mathrm{if}\;1+\left(1-q\right)x>0,\\ 0, & \mathrm{otherwise}. \end{array}\right. \end{array}$$

It can be shown that the traditional exponential function (exp) is given by the limit:

(8) $$\exp\left(x\right)=\lim_{q\to 1}\exp_{q}\left(x\right).$$

## 3. Proposed q-Sigmoid Functions

We built our proposal from the *q*-exponential function which, in turn, is derived from the PME and Tsallis entropy formalism (Section 2). In this context, we suppose a grayscale image $I:D\to \left[0,L\right]$, where $D\subset R^{2}$ is the image domain and a specific region in *D* with an average luminance value β and standard deviation α. With the purpose of contrast enhancement, we consider the sigmoid transformation given by

(9) $$I_{1}\left(I;\beta ,\alpha ,\lambda \right)=\frac{2}{1+\exp\left(\lambda \left(\frac{\left|I-\beta \right|}{\alpha }\right)\right)}.$$

**Figure 1:**
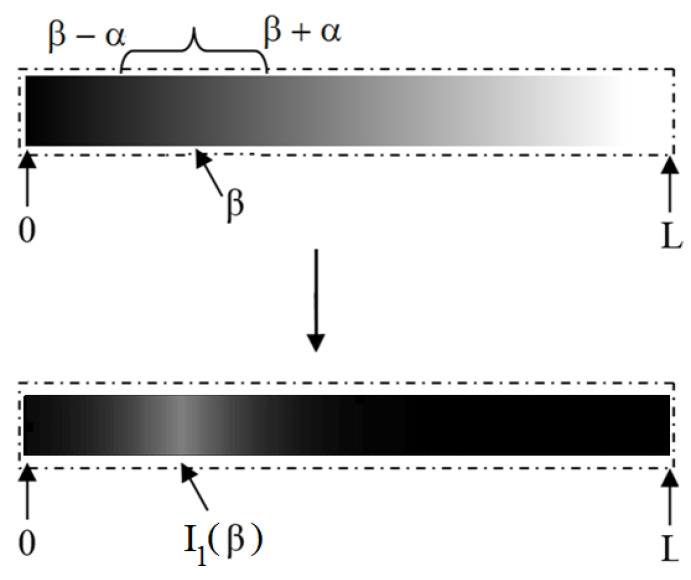
An example of the luminance transformation obtained by Equation (9) with the domain 0≤I(x,y)≤L and parameters β and α indicated. The upper row shows the input image while the bottom shows the obtained result with an enhancement of the region nearby $I_{1}\left(\beta ,\alpha ,\lambda \right)$.

Figure 1 shows a schematic example of the use of parameters β and α in Equation (9). In this figure, we indicate the β value in the input image as well as the range, defined through the parameter α, around the luminance given by β. We can notice that the luminance nearby β was mapped close to 1, while outside the rage [β−α,β+α], it became darker. In this way, we get an enhancement of the target region, of which the pixel intensities fall in the range [β−α,β+α] in the transformed field $I_{1}$.

On the other hand, we get interesting enhancement effects of values nearby β by using another sigmoid-like function defined as

(10) $$\begin{array}{c} I_{2}\left(I;\beta ,\alpha ,\lambda \right)=\left\{\begin{array}{cc} \frac{1}{1+\exp\left(-\frac{\lambda }{\left(\frac{\left|I-\beta \right|}{\alpha }\right)}\right)}, & if\;\;I\neq \beta ,\\ 1, & otherwise. \end{array}\right. \end{array}$$

Following the idea of non-extensive systems for natural as well as medical images [22,27,28,29], in this work, we propose an extended version of Equations (9) and (10), called here as *q*-sigmoid functions, which are defined based on the *q*-exponential function, given by Equation (7) in the forms bellow.

- *q*-Sigmoid for q<1:
  (11) $${\tilde{I}}_{1}\left(I;\beta ,\alpha ,\lambda ,q\right)=\frac{2}{1+{\left[1+\lambda \left(1-q\right)\left(\frac{\left|I-\beta \right|}{\alpha }\right)\right]}^{\frac{1}{1-q}}},$$
- *q*-Sigmoid for q>1:
  (12) $$\begin{array}{c} {\tilde{I}}_{2}\left(I;\beta ,\alpha ,\lambda ,q\right)=\left\{\begin{array}{cc} \frac{1}{1+{\left[1+\lambda \left(1-q\right)F\left(I\right)\right]}^{\frac{1}{1-q}}}, & \mathrm{if}\;\;I\neq \beta \\ 1 & \mathrm{otherwise} \end{array}\right., \end{array}$$
  where
  $$F\left(I\right)=-\frac{1}{\left(\frac{\left|I-\beta \right|}{\alpha }\right)}.$$

Using the fact that, in the limit, q→1 non-extensive expressions are reduced to extensive ones, and applying usual limit properties, it is straightforward to show that

(13) $$\lim_{q\to 1}{\tilde{I}}_{1}\left(I;\beta ,\alpha ,\lambda ,q,\right)=I_{1}\left(I;\beta ,\alpha ,\lambda \right),$$

and

(14) $$\lim_{q\to 1}{\tilde{I}}_{2}\left(I;\beta ,\alpha ,\lambda ,q\right)=I_{2}\left(I;\beta ,\alpha ,\lambda \right),$$

which prove that *q*-sigmoids are extensions of sigmoid functions.

The idea behind the use of *q*-sigmoids rather than sigmoid functions is motivated by the fact that *q*-sigmoids have the extra non-extensive parameter to control the curve’s profile. Therefore, one can better customize filters to each class of applications by tuning *q* besides β, α, and λ parameters. This idea has been used in several fields of applications, mainly in image processing and computer vision applied to medical areas [22,27,29].

**Figure 2:**
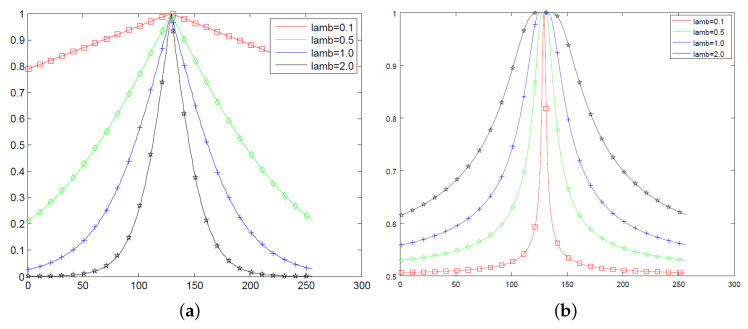
(**a**) The Sigmoid function profile for different λ in Equation (9); (**b**) the modified sigmoid function given by Equation (10).

**Figure 3:**
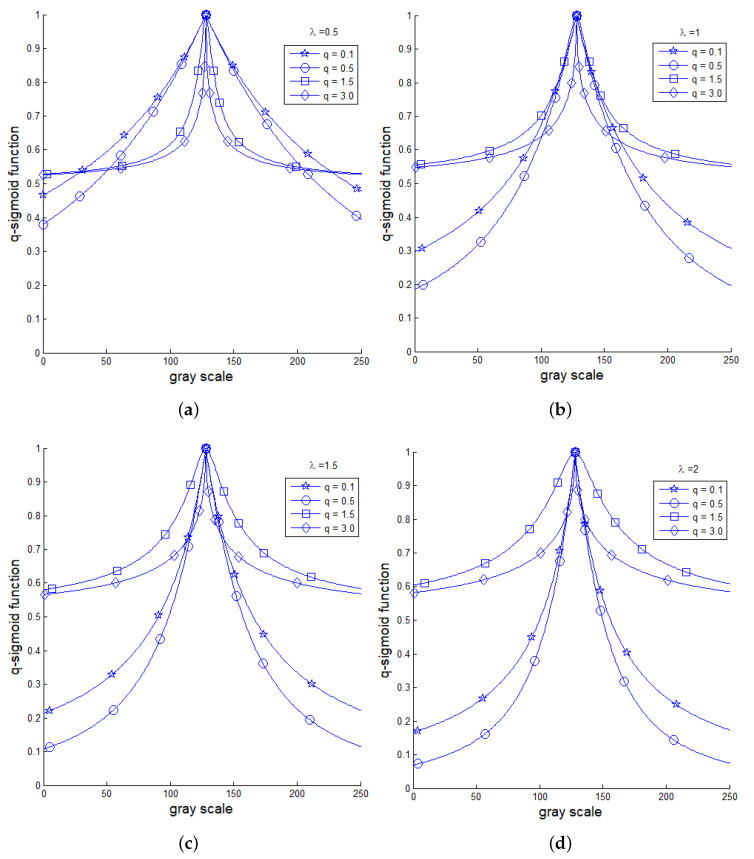
A comparison of *q*-sigmoid profiles of Equations (11) and (12): (**a**) λ=0.5; (**b**) λ=1.0; (**c**) λ=1.5; and (**d**) λ=2.0.

It is interesting to compare the profile of sigmoid and *q*-sigmoid functions under the variation of parameters, such as λ. For this task, we consider some values inside the interval 0≤q≤3. Also, we set I∈[0,255], β=128, and α=30 and built Figure 2 that allows a comparison of the behavior of Equations (9) and (10) for $\lambda \in \left\{0.1,0.5,1.0,2.0\right\}$. Also, Figure 3 shows the profiles of Equations (11) and (12) for $q\in \left\{0.1,0.5,1.5,3.0\right\}$ and $\lambda \in \left\{0.5,1.0,2.0\right\}$.

The visual analysis of Figure 2 and Figure 3 allows us to guess that

(i) Both sigmoid and *q*-sigmoid functions have global maximum at I=β.
(ii) Equation (12) seems to have an asymptotic behavior for large values of *I*.
(iii) The slope is given by
  (15) $$\lim_{I\to \beta_{\pm }}\frac{df}{dI},$$
  where $f\in \left\{I_{1},I_{2},{\tilde{I}}_{1},{\tilde{I}}_{2}\right\}$ could give information about the decay of the corresponding expressions nearby the intensity I=β.
(iv) The limit value in Equation (15) is highly influenced by λ if $f=I_{1}$.
(v) We shall analyze Equation (15) to get the way in which the *q* value influences the decay for $f\in \left\{{\tilde{I}}_{1},{\tilde{I}}_{2}\right\}$ and analogously for expression $I_{2}$ with respect to λ.
(vi) Equation (10) has a null derivative with respect to *I* at I=β.

Property (i) is a direct consequence of Equations (9)–(12), and it is true for the whole family of considered sigmoid and *q*-sigmoid functions. Property (ii) can be verified by observing that

(16) $$\lim_{I\to \infty }{\tilde{I}}_{2}\left(I;\beta ,\alpha ,q\right)=1/2.$$

The other points will be studied in the next section through the derivative of sigmoid and *q*-sigmoid functions.

## 4. Analysis of the Derivatives

For simplicity, we will consider the expressions only for I≥β. The case I<β is analogous with a change of signal only due to the symmetries of the target expressions with respect to I=β.

- Derivative of sigmoid in Equation (9) for I>β:
  (17) $$\frac{dI_{1}}{dI}=-2\left(\frac{\lambda }{\alpha }\right){\left(1+\exp\left[\lambda \left(\frac{I-\beta }{\alpha }\right)\right]\right)}^{-2}\left(\exp\left[\lambda \left(\frac{I-\beta }{\alpha }\right)\right]\right).$$
- Derivative of sigmoid in Equation (10) for I>β:
  (18) $$\frac{dI_{2}}{dI}=-\frac{\lambda }{\alpha }{\left(\frac{I-\beta }{\alpha }\right)}^{-2}{\left(1+\exp\left(-\frac{\lambda }{\left(\frac{I-\beta }{\alpha }\right)}\right)\right)}^{-1}\exp\left(-\frac{\lambda }{\left(\frac{I-\beta }{\alpha }\right)}\right).$$
- Derivative of *q*-sigmoid (Equation (11)) if q<1 and I>β:
  (19) $$\frac{d{\tilde{I}}_{1}}{dI}=-\lambda \frac{2}{\alpha }{\left(1+{\left[1+\lambda \left(1-q\right)\left(\frac{I-\beta }{\alpha }\right)\right]}^{\frac{1}{1-q}}\right)}^{-2}{\left[1+\lambda \left(1-q\right)\left(\frac{I-\beta }{\alpha }\right)\right]}^{\frac{q}{1-q}}.$$
- Derivative of *q*-sigmoid, given by Equation (12), if q>1 and I>β:
  (20) $$\frac{d{\tilde{I}}_{2}}{dI}=-\frac{\lambda }{\alpha }{\left(1+{\left[1+\lambda \left(1-q\right)F\left(I\right)\right]}^{\frac{1}{1-q}}\right)}^{-2}{\left[1+\lambda \left(1-q\right)F\left(I\right)\right]}^{\frac{q}{1-q}}{\left(\frac{I-\beta }{\alpha }\right)}^{-2}.$$

From Equations (17)–(19), the following properties are straightforwardly verified:

(21) $$\lim_{I\to \beta_{+}}\frac{dI_{1}}{dI}=-\frac{\lambda }{2\alpha };$$

(22) $$\lim_{I\to \beta_{+}}\frac{dI_{2}}{dI}=0.$$

(23) $$\lim_{I\to \beta_{+}}\frac{d{\tilde{I}}_{1}}{dI}=-\frac{\lambda }{2\alpha }.$$

As stated in Property (v) of Section 3, we shall consider the derivative results above to quantify the influence of parameters *q* and λ. From Equation (21), it becomes obvious that the behavior of Equation (9) is highly influenced by λ, as also noticed in the plots of Figure 2a and pointed out in Property (iv). Equation (22) demonstrates Property (vi) which states that the modified sigmoid Equation (10) is smooth at I=β with a null derivative with respect to *I*. Equation (23) shows that the decay of ${\tilde{I}}_{1}$ nearby the intensity I=β is almost insensitive to the *q* variation. To get more information about the decay of ${\tilde{I}}_{1}$ in a neighborhood of I=β, we shall consider the first terms of the Taylor series to approximate Equation (11) as

(24) $${\tilde{I}}_{1}\left(I;\beta ,\alpha \right)\approx 1-\frac{\lambda }{2\alpha }\left(I-\beta \right),$$

Hence, we can compare the first-order approximation in Equation (24) with the *q*-sigmoid through

(25) $$R\left(I;\beta ,\alpha ,\lambda ,q\right)=\frac{{\tilde{I}}_{1}\left(I;\beta ,\alpha ,\lambda ,q\right)}{\left(1-\frac{\lambda }{2\alpha }\left(I-\beta \right)\right)},$$

We experimentally notice that this expression is monotonically increasing with respect to *I*. Moreover, for λ=1, I=(β+α/2)=143, and 0<q<1, we get $R\left(I=143;128,30,q\right)<1.07$ which shows that the (almost) linear behavior observed for $q\in \left\{0.1,0.5\right\}$ in Figure 2c is a general property of Equation (11).

**Figure 4:**
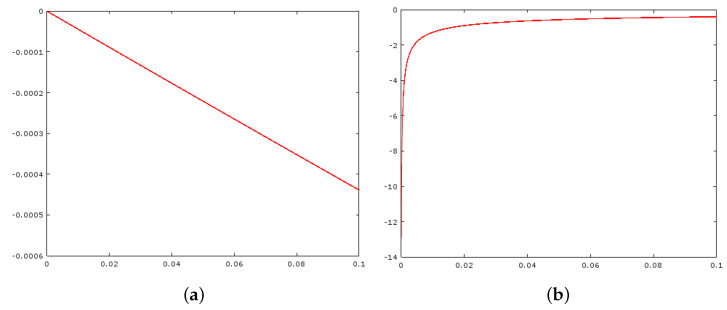
(**a**) The difference Equation (26) for I=128, β=128, α=30, q=1.5, and 0.0001≤ΔI≤0.1; (**b**) The behavior of Equation (26) in I=128 with the same parameters, but q=3.0.

An analogous procedure can be performed by Equation (9). However, such an analysis is not so simple when considering Equation (12) because its derivative with respect to *I* does not have an analytical form for I=β. To get some guess about Equation (15) for I>β, we plot in Figure 4 the difference equation:

(26) $$D\left(I;\beta ,\alpha ,q\right)=\frac{{\tilde{I}}_{2}\left(I+\Delta I\right)-{\tilde{I}}_{2}\left(I\right)}{\Delta I}$$

for I=128, β=128, α=30, $q\in \left\{1.5,3.0\right\}$, and 0.0001≤ΔI≤0.1

The plots in Figure 4 indicate that the derivative of Equation (12) depends on the *q* value, which also agrees with the visual analysis of Figure 2c. From Figure 4a, we could guess that $\lim_{\Delta I\to 0_{+}}D\left(128,\Delta I;128,30,1.5\right)=0$ while $\lim_{\Delta I\to 0_{+}}D\left(128,\Delta I;128,30,1.5\right)\in \left(-14,-12\right)$ which implies a dependence of $d{\tilde{I}}_{2}/dI$ with respect to parameter *q* that cannot be explained with the first-order approximation for all ranges q>1.

## 5. Experimental Results

Before starting the computational experiments, it is worthwhile to state the practical consequences of Properties (i)–(vi) and the theoretical analysis of Section 4. Firstly, due to Property (i), we can assure that functions (9)–(12) highlight the pixel intensities nearby I=β, as pictured in Figure 1. From Equations (21)–(23), we can say that the way the transformed image intensity decays in a neighborhood of I=β is linear (respect to *I*) for Equations (9) and (11) and quadratic (at least) for the modified sigmoid (10). Moreover, in the linear case, it does not depend on the *q* value, and the linear coefficients are proportional to λ and inversely proportional to α. However, the numerical experiments reported in Figure 4 show that the derivative of Equation (20) undergoes a different behavior nearby I=β, which depends on the *q* value besides λ.

In this paper, initially we propose an experimental analysis under artificial data to make easier a quantitative evaluation of the *q*-sigmoid for image enhancement. Then, we repeat the experiments for real images. The implementations were performed using the MATLAB Release 13 facilities. For the artificial data analysis, we built a 2-D Gaussian distribution so that we can manipulate its parameters (mean μ and standard deviation δ). The Gaussian’s amplitude is normalized between $\left[0,1\right]$ so that we can observe its 3-D profile as a grayscale image, creating artificial conditions for the application of the proposed filters.

**Figure 5:**
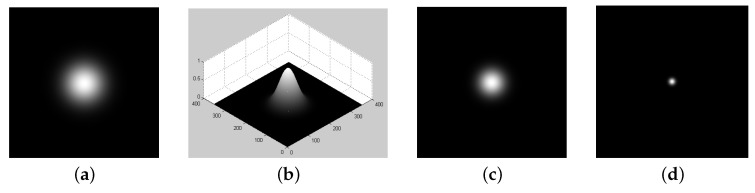
(**a**) The intensity profile of a normalized 2-D Gaussian distribution, with a mean μ=175 and a variance δ=30 in orthogonal view; (**b**) the corresponding 3-D perspective; (**c**) an example of filtering with sigmoid when α=0.02; (**d**) an example of filtering with sigmoid when α=0.01.

With this idea at hand, we can use the Gaussian parameters in order to create severe conditions to improve the contrast and to enhance some regions of interest. For this purpose, we defined as a region to be highlighted the one with an intensity in the interval μ±3×δ. Figure 5 shows this setup, where Figure 5a is the original image with a clear region of interest, and Figure 5b is its corresponding perspective visualization.

### 5.1. Locally Linear Behavior

In this section, we perform computational experiments using the families given by Equations (9) and (11), of which the derivatives at I=β are linear functions with respect to λ (see Equations (21) and (23)). Therefore, we set the parameters β=1.0 and λ=1.0 and vary the α value for both the sigmoid (Equation (9)) and *q*-sigmoid (Equation (11)) functions. Under the scenario of Equations (21) and (23), when α is set to small values (e.g., 0.02 or 0.01), the rate of decrease of the transformed image intensity becomes too high. Consequently, the highlighted region in the output field can be very small compared to the original one (e.g., Figure 5c–d).

Now, we keep the value of β=1.0 and λ=1.0 and set the value of α=0.03 for both the sigmoid and *q*-sigmoid functions. The *q*-sigmoid function was parameterized by taking also q∈{0.7,0.8,0.9,0.95,0.98,0.99,0.999}. We apply the two filters in the image shown in Figure 5a.

The values of *q* were chosen in order to highlight significant behaviors. For instance, for q<0.7, the function *q*-sigmoid gets optimal results for all α values studied here (the case q>1.0 is discussed later). Note that the value q=1.0 corresponds to the traditional sigmoid function, since *q*-sigmoid reduces to sigmoid when *q* tends to 1.0, as explained in Section 3.

For each value of *q*, the *q*-sigmoid filter of Equation (11) was applied on the artificial image of Figure 5a. The same was done for the sigmoid filter in Equation (9). Since each highlighted region may represent a connected set of pixels, a comparison between both results (by sigmoid and *q*-sigmoid) is accomplished by relating the convex-hull of both enhanced and original areas, according to the following equation:

(27) $$Err=\left|1-\frac{A_{1}}{A_{0}}\right|$$

where $A_{1}$ is the convex-hull area of the *q*-sigmoid/sigmoid achieved region and $A_{0}$ is the ground-truth area defined in a Gaussian grayscale profile image by setting μ±3×δ. When Err achieves zero, it means that *q*-sigmoid/sigmoid filtering gets the optimal performance.

**Figure 6:**
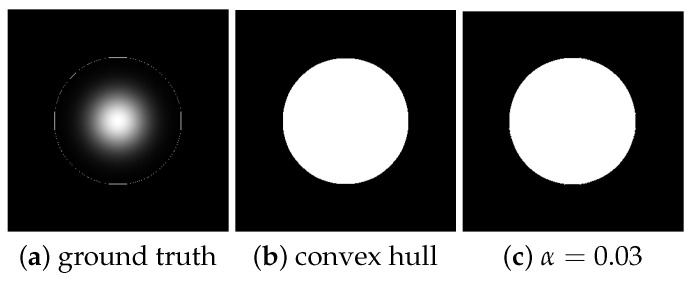
(**a**) The intensity profile of a 2-D Gaussian distribution in orthogonal view with the corresponding convex-hull points indicating the ground truth area; (**b**) the corresponding convex-hull filled area; and (**c**) the convex-hull-filled area achieved when α=0.03, β=1.0, λ=1.0, and q=1.0.

Table 1 shows the error for each value of *q* cited above. Figure 6 shows the corresponding visual result when q=1.0. Figure 6a shows the original Gaussian grayscale profile but now with a circle around indicating the convex hull of the ground truth area. Figure 6b is the corresponding filled area, and Figure 6c is the achieved area after sigmoid filtering. The visually imperceptible difference (confirmed in Table 1 for q=1.0) between areas of Figure 6b,c indicates that this was a good performance of filtering approach.

**Figure 7:**
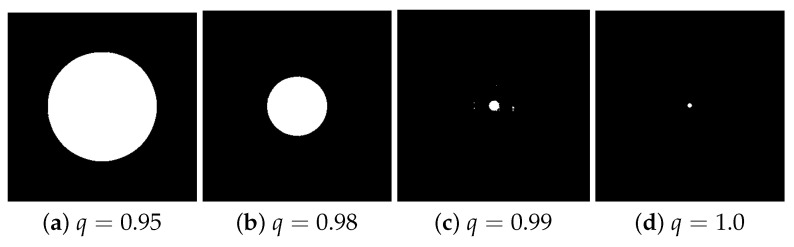
(**a**) The convex-hull area achieved when q=0.95; (**b**) the convex-hull area achieved when q=0.98; (**c**) the convex-hull area achieved when q=0.99; and (**d**) the convex-hull area achieved when q=1.0.

On the other hand, in the more extreme case, we set α=0.01, β=1.0, and λ=1.0 and apply the *q*-sigmoid function for all values of *q* in our set up. Under this new scenario, the value of α is too small, severely hampering the search by the region of interest. To allow the comparison with the results of Table 1 and Figure 6, we report in Table 2 the Err measure found for each *q* value and show in Figure 7 the corresponding visual results.

Now, according to Table 2, some values of *q* are more sensitive to the variation of α and present a low performance for the same input of Figure 5a. In Figure 7, we present four convex hulls found for three *q*-sigmoid filters (when q∈{0.95,0.98,0.99}) and the sigmoid one (when q=1.0). For all other *q* values, including q≥1.0, under α=0.01, the *q*-sigmoid filtering behavior was optimal.

For completeness, we show in Table 3 the same results observed in the Table 1 and Table 2 but add the results for other values of α∈{0.01,0.015,0.02,0.025,0.03}. As said before, for α≥0.03, it is no longer extreme cases and both filters achieve the same optimal performances, so these cases are not shown here.

**Figure 8:**
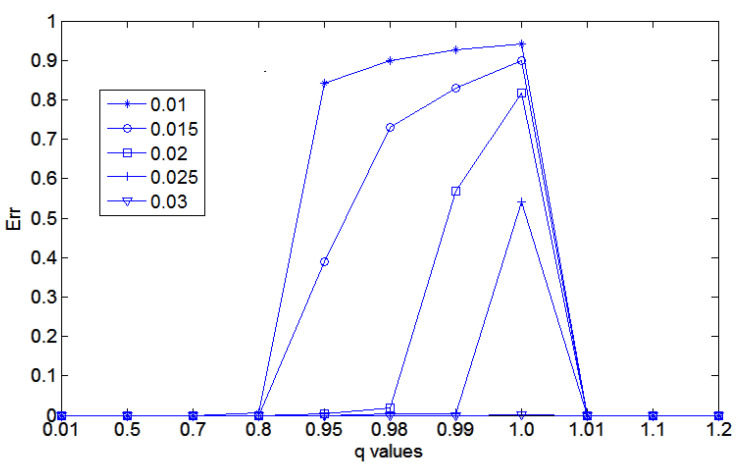
The overview performance under increasing α values. Each curve corresponds to an α value used for an enhancement filtering of the artificial grayscale 3-D Gaussian profile. We also show Err for q>1.0.

Finally, Figure 8 shows the overall observed behavior in Table 3. In this figure, we can clearly see that as the α value increases (shown inside the small box), the value of Err also decreases for all values of *q*. However, for each value of α, the value of q=1.0, the traditional sigmoid function presents the worst performance. In this figure, we also show results for q>1.0.

Clearly, Table 3 and Figure 8 show the superiority of the *q*-sigmoid filtering over the traditional sigmoid one to highlight images in extreme conditions when the range of values around β are known to be small.

### 5.2. Generalized Behavior

Now, we shall analyze results using Equation (12) that depends on the *q* value besides λ. We follow the same methodology of previous sections and apply the proposed enhanced technique over the input image pictured in Figure 5a.

Initially, we set the value of β=1.0, α=0.03, and λ=1.0 for both sigmoid (Expression (10)) and *q*-sigmoid functions (Equation (12)) and applied the filters in the image shown in Figure 5a. The *q*-sigmoid function was applied with values of q∈{1.01,1.1,1.2}. A comparison between both results was accomplished by computing Equation (27). However, the errors for all considered value of *q*, when β=1.0 and α=0.03 were zero.

On the other hand, in the extreme case, we set α=0.01 and applied the *q*-sigmoid function for all values of q∈{1.01,1.1,1.2}. Under this new scenario, the value of α was too small, severely hampering the search by the region of interest.

Table 4 shows the performance of sigmoid (q=1) and *q*-sigmoid for increasing values of α and *q*. Note that Err for values of q>1.0 tends to zero for all α ranges.

### 5.3. Experiments with Ultrasound Images

The tests in artificial images indicate how *q*-sigmoid functions behave for image enhancement regarding their parameters. We conclude that *q*-sigmoids defined by Equations (11)–(12) achieve the better performance in the enhancement tests using Figure 5a. The *q*-sigmoid results tend to highlight with a higher contrast the region of interest from the background, as shown when comparing the synthetic experiments of Figure 6 and Figure 7. We would like to exploit this property in applications involving medical images.

**Figure 9:**
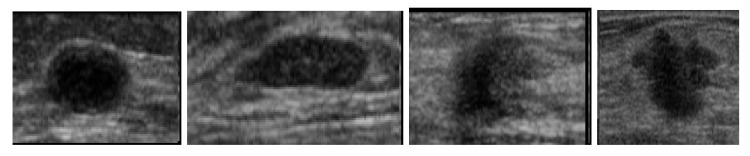
Four examples of breast ultrasound images with lesions in a darker gray scale nearby the center of the images.

Besides the influence of noise and low contrast, the morphology of regions of interest is also a factor that hampers the assessment of filters considered to highlight the target information. For instance, ultrasound images as seen in Figure 9 are heavily corrupted by speckle noise, besides having target objects not clearly defined. Under such conditions, the region of interest (lesion) can merge with the background, making difficult further segmentation operations.

Thus, the greatest challenge faced by image enhancing methods automatically isolates the lesion from its background, reducing the interference of noise and artifacts in further operations.

A well-known method for image enhancement is the slicing, which consists of mapping the original intensity range within a small track [30]. The most popular mapping is one that generally excludes the lower and higher intensity ranges of the original image. The justification for this slicing method is that the human visual system is much more sensitive to medium intensity ranges than low and high intensities. Besides slicing, histogram equalization also improves the visual contrast of such images, but it does not mean that they facilitate the automatic extraction of the lesion. One reason is because these two popular methods, although they tend to produce more visually pleasing results, also enhance noise, artifacts, and contrast between unwanted areas. In order to compare the slicing and histogram equalization results with the ones obtained with *q*-sigmoids, we use a statistical measure that quantifies the difference in intensity between two images, in our case, the original image (*X*) and the output one (*Y*) obtained by the considered methods. Specifically, we apply the absolute mean brightness error (AMBE), which can be calculated through the absolute difference between the mean intensities of *X* and *Y*:

(28) AMBE=|E(X)−E(Y)|,

where E(·) denotes the statistical mean.

Equation (28) clearly shows that AMBE is designed to detect excessive change in the brightness. In current practice, a lower AMBE implies that the original brightness is better preserved and, hence, that the image *Y* should yield a better enhancement of the region of interest.

**Figure 10:**
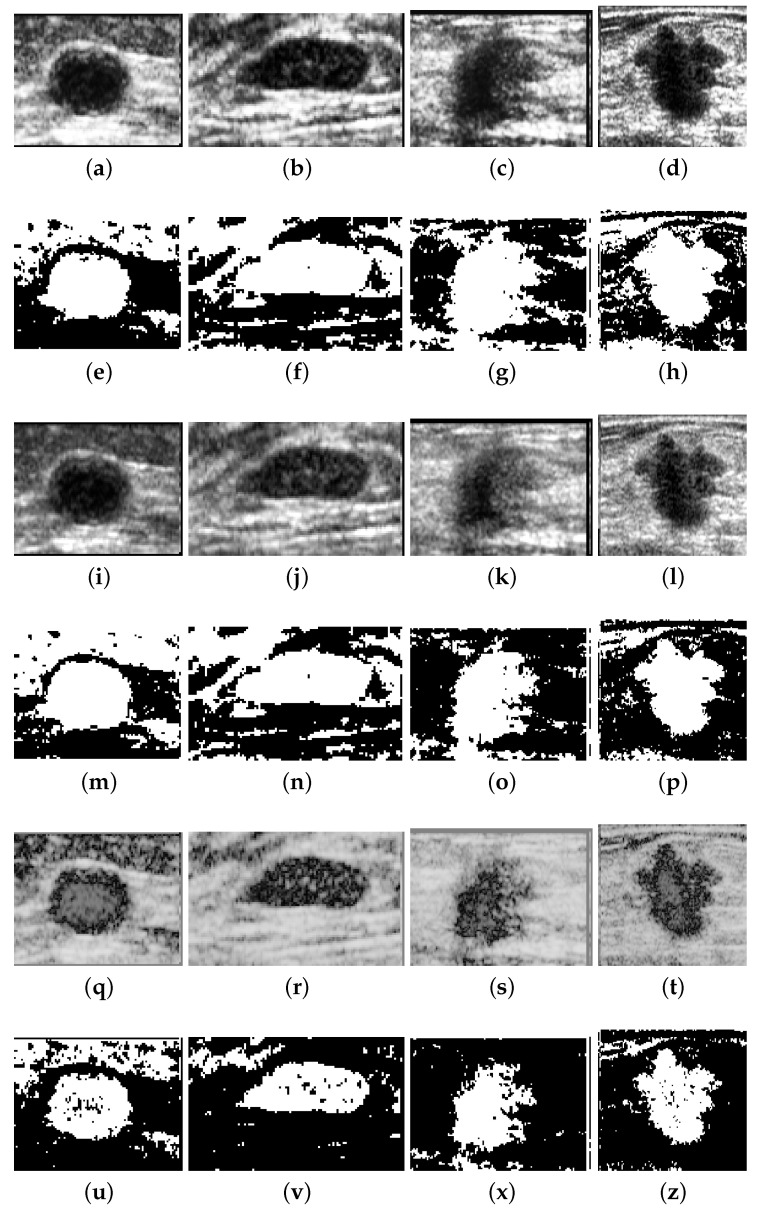
The results of application of enhancing methods over the four images of Figure 9. (**a**–**d**) The results of histogram equalization. (**e**–**h**) The segmentation after histogram equalization. (**i**–**l**) The slicing map results. (**m**–**p**) The segmentation after slicing. (**q**–**t**) The results obtained with *q*-sigmoid when β=0.15, α=0.03, λ=1.0, and q=2.0 in Equation (12). (**u**–**z**) The segmentation results after preprocessing with *q*-sigmoid.

Figure 10 allows a visual comparison between the results of the histogram equalization, slicing, and *q*-sigmoid given by Equation (12) when applied to the four images in Figure 9. In these examples, it can be noted from Figure 10a–d and Figure 10i–l that the histogram equalization method and slicing-based technique obtain a better visual result than the proposed method (Figure 10q–t). However, the output of *q*-sigmoid has a much stronger contrast, clearly facilitating the isolation of region of interest from the background as we can check in the images of Figure 10q–t. In Figure 10u–z, we show the segmentation results obtained by preprocessing the original images with *q*-sigmoid and entering the obtained result in the Otsu thresholding algorithm [24]. If compared with the segmentation results obtained after preprocessing with counterpart techniques (Figure 10e–h and Figure 10m–p), we can visually confirm that the lesion segmentation, in general, is better extracted after a *q*-sigmoid enhancement. Specifically, Figure 10e–m,u shows equivalent results in the sense that the region of interest is isolated from the surrounding object. In this line, the results in Figure 10v,x favor a *q*-sigmoid against the counterpart methods (Figure 10f,n,g,o) once there are no bridges between the target object and its neighborhood. The segmentation presented in Figure 10h,p,z can be considered equivalent ones.

In order to obtain a quantitative comparison, we apply the AMBE, formally described by Equation (28). The test database contains 250 ultrasound images of breast cancer, 100 malignant, and 150 benign. The images in Figure 9 are four samples of this database. The images are processed by the considered methods, and Equation (28) was applied for a subsequent analysis.

**Figure 11:**
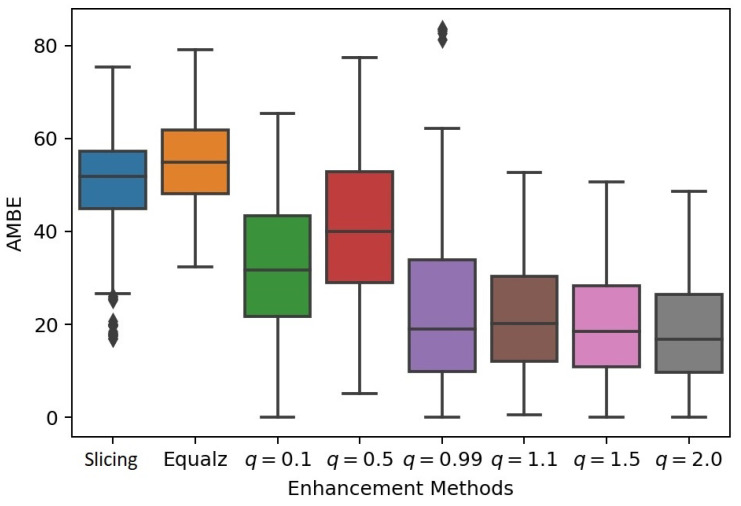
An overview performance under histogram equalization (first left block), slicing (second block), and *q*-sigmoids (Equations (11) and (12)) with increasing *q* values (third to eighth block). Each block stands for a mean and standard deviation of absolute mean brightness error (AMBE) measure in the vertical axis.

For the proposed method, we set β=0.15, α=0.01 and λ=1.0, and we varied the *q* value in *q*-sigmoid as q∈{0.1,0.5,0.999,1.1,2.0}. For each of the 250 images of the database, the three methods were then applied. Figure 11 allows the comparison of the performance of classical methods (equalization and slicing) and the proposed method with the five *q* values cited before, given a total of eight comparative instances for each sample. The box-plot of Figure 11 clearly shows that the proposed method outperforms the other two (histogram equalization and slicing-mapping) with any value of *q* in the *q*-sigmoid function.

It can be seen that the best performance was obtained with the value of q=2.0. On the other hand, although for the value of q=0.999 we have obtained a better result than the two other traditional methods, it is the configuration that generates the highest standard deviation.

### 5.4. *q*-Sigmoid and CNN Segmentation

This section discusses the *q*-sigmoid efficiency as a preprocessing step in CNN-based image segmentation pipelines. To demonstrate this idea, we used two databases in our experiments. The first one is a 256×256 artificial image database, generated through the Gaussian function shown in Figure 5a. In order to build such a database, we add to the gray scale image in Figure 5a different Gaussian noise levels, generating 100 images with signal to noise ratio (SNR) from SNR=0 to SNR=100. The second grayscale image database is composed by the 250 breast cancer ultrasound images used in Section 5.3. Examples of these images can be seen in Figure 9, where the central part of the images represent the lesion (region of interest). For both databases, we randomly separate 70% images for training and the remaining ones for test, using a cross-validation strategy for CNN training.

The databases were tested on two CNNs with the same architecture: 3 convolutional, 2 max pooling, and 1 transpose layer. The convolutional layers use Relu, and the transpose applies the linear activation functions. The networks are named $CNN_{A}$ and $CNN_{U}$, of which the purpose is to segment the input images (artificial for $CNN_{A}$ and ultrasound for $CNN_{U}$) by separating the region of interest from its background.

**Figure 12:**
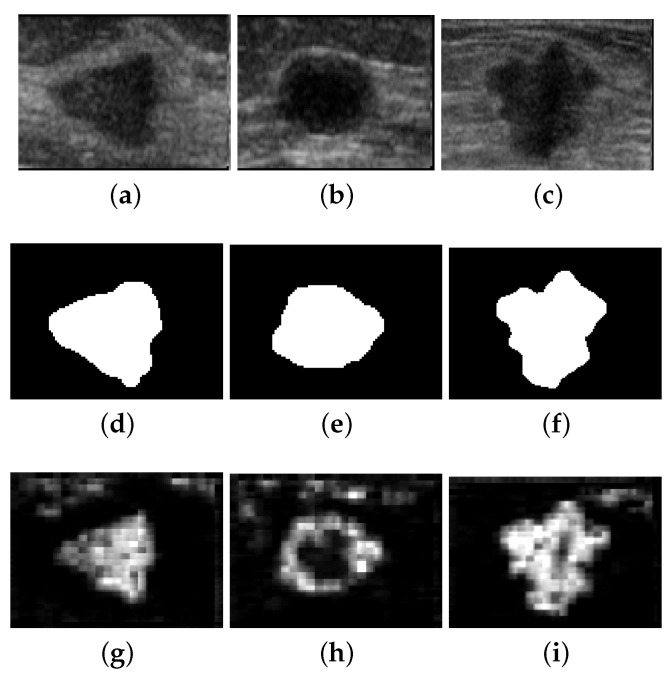
(**a**–**c**) Original ultrasound images. (**d**–**f**) The segmentation ground-truth. (**g**–**i**) The segmentation obtained by CNN after filtering by *q*-sigmoid when β=0.15, α=0.03, λ=1.0, and q=0.1 in Equation (12).

The segmentation quality measure used in this work compares the CNNs prediction with a ground-truth. For the artificial database, the ground-truth is the image shown in Figure 5a. In the case of ultrasound images, they were manually segmented by an expertise to build the ground-truth as pictured in Figure 12.

Therefore, we fed the trained CNNs with a test image and computed the similarity between its segmentation result (say *A*) and the ground-truth (say *G*) by expression:

(29) $$S\left(A,G\right)=\frac{1}{2}\left(\frac{\#\left(A\cap G\right)}{\#\left(A\cup G\right)}+\frac{\#\left[\left(G⊕B-G\right)\cap ∼A\right]}{\#\left(G⊕B-G\right)}\right),$$

where # means the number of pixels, ⊕ is the dilation operation, *B* is the canonical 3×3 structuring element [31], and ∼A means the negative of image *A*. The above expression is motivated by the fact that, in the case of ultrasound images, the $CNN_{U}$ might extract other objects with similar texture patterns besides the region of interest, as observed in Figure 12g–i. If those regions are disconnected in the segmentation result, it is easy to isolate them in a further process of information extraction. In this case, the ratio $\#\left[\left(G⊕B-G\right)\cap ∼A\right]/\left(\#\left(G⊕B-G\right)\right)$ is approximately one, indicating that the $CNN_{U}$ isolates the region of interest. Otherwise, this ratio falls in the interval $\left[0,1\right]$, penalizing the similarity measure because the lesion is connected with other regions in the segmented image.

**Figure 13:**
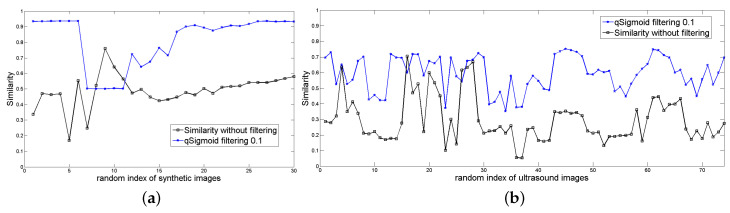
(**a**) A comparative performance of the $CNN_{A}$ output without filtering (black line) and with preprocessing by *q*-sigmoid (q=0.1, β=1.0, α=0.8, γ=1.0 in Equation (11)), shown by the blue line. The vertical axis is the similarity calculated according to Equation (29), and the horizontal axis lists the synthetic images with different noise levels randomly chosen; (**b**) The analogous result for $CNN_{U}$ with q=0.1, β=0.6, α=0.2, and γ=1.0 in Equation (11).

Therefore, we apply the *q*-sigmoid filter to each image before entering the CNN and computing Equation (29) to measure the quality of the segmentation obtained. Figure 13a shows the $CNN_{A}$ efficiency for the segmentation of the synthetic database (black line) and its filtered version (blue line). Figure 13b reports an analogous result for $CNN_{U}$.

From Figure 13a,b, we notice that the application of *q*-sigmoid as a preprocessing step, in general, improves the segmentation results for both $CNN_{A}$ and $CNN_{U}$, although this fact is more evident in the case of artificial images because these images are less challenging than the ultrasound ones to be segmented.

## 6. Discussion

This paper describes the applicability of the proposed *q*-sigmoid functions, defined by Equations (11) and (12) in breast ultrasound images, which are images highly affected by speckle noise. Moreover, tests were performed with artificial images, under controlled parameterization demonstrating the superiority of *q*-sigmoids against their classical sigmoid versions.

It should be emphasized that our goal is not to improve global contrast but to highlight a specific region of interest, as in the case of the lesions in the breast ultrasound images of Figure 9. The box-plot of Figure 11 clearly shows that the performance of the proposed *q*-sigmoid function overcomes the techniques of slicing and equalization in this task. Clearly, there is a decay of the AMBE measure as the *q* value increases, indicating that, although the ideal value of *q* has not been pre-calculated, an acceptable range is easily defined, that is, the proposed *q*-sigmoid function produces a good performance for a large range of *q* values.

It is also noticed in the synthetic experiments of Section 5.1 the great influence of the values of *q* in relation to function decay. Thus, although this sensitivity is large, due to the overall good performance for a wide range of *q* values, this behavior does not affect negatively the final result.

The results of Section 5.3 and Section 5.4 indicate that the *q*-sigmoid preprocessing can simplify segmentation stages if used in pipelines, such as the ones of CAD (Computer Aided Diagnosis) systems. This is the main factor that highlights the proposed functions as promising strategies to be used in computational systems of image analysis even considering recent approaches based on CNNs. In fact, Figure 10 and Figure 13 show some results, indicating that the *q*-sigmoid can improve the segmentation obtained by Otsu and CNN in this application.

**Figure 14:**
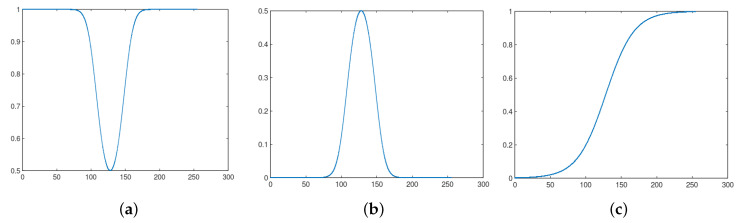
(**a**) The profile of fractional logistic function with $D_{max}=1$, C=1, b=2, k=0.05, and $I_{0}=128$; (**b**) Negative of the fractional logistic function; (**c**) The S-shaped logistic function obtained by setting b=1 and keeping the other parameters unchanged.

Also, we shall include some comments about the relationship between our work and the fractional logistic function written as

(30) $$D\left(I\right)=\frac{D_{max}}{1+C\exp\left(-{\left(k\left(I-I_{0}\right)\right)}^{b}\right)},$$

where $D_{max},C,k,b>0$ with 0.5<b<3.0 [32,33]. We can demonstrate that the derivative of Equation (30) with respect to *I* is zero in $I=I_{0}$ and that this point is a local minimum. For b=2, named a quadratic logistic function in Reference [33], we can verify that $\lim_{I\to +\infty }D\left(I\right)=\lim_{I\to -\infty }D\left(I\right)=D_{max}$. Moreover, Equation (30) does not have other critical point different from $I=I_{0}$. Consequently, Equation (30) has the profile given by Figure 14a if b=2. In Figure 14b, we show the profile of $f\left(I\right)=1-D\left(I\right)$, which is a bell-shaped function. Therefore, it resembles the plots in Figure 2b, where we have $dI_{2}/dI=0$ if I=β, as shown by Equations (18) and (22).

Consequently, a local analysis nearby $I_{0}=\beta$ shows an equivalent behavior between $I_{2}\left(I\right)$ in Equation (10) and $f\left(I\right)=1-D\left(I\right)$. Far from I=β, we can adjust the parameters α and λ such that we obtain $I_{2}\left(I\right)\approx f\left(t\right)$. Hence, the quadratic fractional logistic function and the sigmoid in Equation (10) generate (almost) equivalent enhancement results. Consequently, once *q*-sigmoid functions surpass the sigmoid one, we expect the same conclusion regarding the quadratic fractional logistic function.

On the other hand, if b=1 in Equation (30), we get an S-shaped profile discussed in References [32,33], like the one pictured in Figure 14c. Therefore, in this case, the behavior of the logistic function family is very different from the bell-shaped *q*-sigmoid functions represented in Figure 3.

However, in another way, from the result of Equation (8), we can write

(31) $$\lim_{q\to 1}{\left[1+\left(1-q\right)\left(-{\left(kt\right)}^{b}\right)\right]}^{1/\left(1-q\right)}=\exp\left(-{\left(kt\right)}^{b}\right).$$

Therefore, we can generalize Equation (30) as

(32) $$\tilde{D}\left(I\right)=\frac{D_{max}}{1+C{\left[1+\left(1-q\right)\left(-{\left(kt\right)}^{b}\right)\right]}^{1/\left(1-q\right)}},$$

and study its behavior for q≠1. We intend to perform such task as future works.

Regarding the parametrization of *q*-sigmoids, we shall perform some comments once we set the parameters β,α,λ, and *q*. The parameters β and α define the interval $\left[\beta -\alpha ,\beta +\alpha \right]$ that we want to highlight in the output image, as pictured in Figure 1. Therefore, these values are set using the knowledge about the regions of interest. In the case of Equation (11) of the material, the parameter λ controls the decay of the image intensity nearby I=β, as noticed in Equation (24). If we want to sharpen the highlighted region, we must increase it. Otherwise, we shall set it smaller. In the case of images with a gray scale intensity in the interval $\left[0,1\right]$, a systematic procedure would be to start with λ=1 and then to make it larger or smaller depending on the effect the user wants. The setting of parameter *q* is more involved, and in general, it is performed by defining a sequence of *q*-values and analyzing the results to realize the best one. One suggestion is to take some values smaller and larger than q=1 besides the q=1 itself that corresponds to the sigmoid functions. We proceed in this manner to generate Figure 11 of the submitted manuscript.

Moreover, we would like to comment another approach that we shall analyze in further works. We can transform the intensity values of the input image such that the histogram of the output field matches the solution of the PME discussed in Section 2. Formally, we suppose a random variable u≥0 with a probability density $p_{1}\left(u\right)$ given by the histogram of the input image. Then, inspired by the histogram equalization approaches [34], we transform the variable *u* in another random variable v≥0 such that its probability density $p_{2}\left(v\right)$ is given by the solution of the PME. To perform this task, it is just a matter of defining the functions:

(33) $$\int_{0}^{u}p_{1}\left(x\right)dx=F_{1}\left(u\right),\;\int_{0}^{v}p_{2}\left(y\right)dy=F_{2}\left(v\right),$$

and imposing that the variable *v* must satisfy the constraint $F_{2}\left(v\right)=F_{1}\left(u\right)$, which gives

(34) $$v\left(u\right)=F_{2}^{-1}\left(F_{1}\left(u\right)\right).$$

Now, the idea is to solve the problem:

(35) $$\arg\min_{\beta ,\alpha ,\lambda ,q}\left|v\left(u\right)-\tilde{I}\left(u;\beta ,\alpha ,\lambda ,q\right)\right|$$

and to seek for parameters $\left(\beta ,\alpha ,\lambda ,andq\right)$ such that the *q*-sigmoid $\tilde{I}\left(u,\beta ,\alpha ,\lambda ,andq\right)$, wherever ${\tilde{I}}_{1}$ or ${\tilde{I}}_{2}$ are, approximates *v* defined by Equation (34). In this manner, we strengthen the association of the *q*-sigmoid functions with Tsallis entropy because the random variable *v* has a probability density $p_{2}\left(v\right)$ that is the solution of the Tsallis PME. On the other hand, we can generate a systematic procedure to set the model parameters in the *q*-sigmoids.

Moreover, it is important to emphasize the consequences of the *q*-calculus and *q*-analysis, which are subproducts of the Tsallis formalism to the signal processing area. In this way, in Reference [35,36], the *q*-deformation of known functions, introduced in the *q*-calculus [37], are considered that allow the consideration of some modifications in known functional transforms (wavelets and Gabor transform, for instance) in order to discuss the performance of image processing techniques on noisy objects in the frequency domain. Also, *q*-Gaussian and difference-of-q-Gaussian kernels have been used as generalizations of the classical counterparts, with outstanding results for noise reduction and high-pass filtering (see Reference [38] and references therein).

## 7. Conclusions

This paper introduced the *q*-sigmoid functions as spatial filters for image enhancing in order to isolate the region of interest. The main objective was to highlight a particular region and not visually improve the image through the contrast enhancement. The *q*-sigmoid functions are generalizations of the well-known sigmoid families, obtained when setting q=1 in Equations (11) and (12).

Tests were carried out with synthetic images when the proposed method outperformed the sigmoid functions under a range of parameter values that leave the scenario in extreme conditions. When the α value is extremely low, the *q*-sigmoid functions always get better results than the sigmoid family, especially for values of q≠1.0. The method was also applied to ultrasound breast cancer images, which are severely affected by the speckle noise, low contrast, and subjective boundary. In this case, the proposed method was also compared with two other known methods of contrast enhancement (histogram equalization and slicing) using the AMBE, which allows the measurement of the contrast level of the transformed image against the level of contrast of the original one. The proposed method achieved better results in terms of AMBE than the counterpart ones for q∈{0.1,0.5,0.999,1.1,2.0}, suggesting that the *q*-sigmoids are promising for region enhancement and can be used in CAD systems for this type of image. In particular, the Otsu and CNN segmentation results obtained when preprocessing ultrasound images with *q*-sigmoids indicate the capabilities of the functions in segmentation pipelines.

As further works, we intend to apply *q*-sigmoid functions for other image modalities to emphasize the contribution of our proposal as a general technique for image enhancement. Also, we shall compare *q*-sigmoids with the generalization of fractional logistic functions (Equation (32)) and to implement a scheme to solve problem (Equation (35)).

## Figures and Tables

**Table 1:** Err demonstration under a range of *q* values with q<1.0, α=0.03, β=1.0, and λ=1.0.

*q*	0.7	0.8	0.9	0.95	0.98	0.99	1.0
Err	0.0	0.0	0.0	0.00	0.0	0.00	0.0001

**Table 2:** Err demonstration under a range of *q* values around q=1.0 and α=0.01.

*q*	0.7	0.8	0.9	0.95	0.98	0.99	1.0
Err	0.0	0.0	0.0	0.0052	0.8412	0.8992	0.9266

**Table 3:** Err demonstration for q<1.0, β=1.0, λ=1.0, and some α values.

Err∖q	0.7	0.8	0.9	0.95	0.98	0.99	1.0
α=0.01	0.0	0.0	0.0	0.0052	0.8412	0.8992	0.9426
α=0.015	0.0	0.0	0.0	0.0	0.3893	0.7298	0.8996
α=0.02	0.0	0.0	0.0	0.0	0.0037	0.0182	0.8173
α=0.025	0.0	0.0	0.0	0.0	0.0000	0.0027	0.5422
α=0.03	0.0	0.0	0.0	0.0	0.0000	0.00	0.001

**Table 4:** Err demonstration under a range of *q* values around q=1.0 and a range of α values.

Err∖q	1.0	1.001	1.1	1.2
α=0.01	0.0	0.00	0.00	0.0
α=0.015	0.0	0.00	0.00	0.0
α=0.02	0.0	0.00	0.00	0.0
α=0.025	0.0	0.00	0.00	0.0
α=0.03	0.0	0.00	0.00	0.0

## References

[1] Imtiaz M.S., Wahid K.A. (2015). Color Enhancement in Endoscopic Images Using Adaptive Sigmoid Function and Space Variant Color Reproduction. Comp. Math. Methods Med..

[2] Foruzan A.H., Chen Y.W. (2015). Improved segmentation of low-contrast lesions using sigmoid edge model. Int. J. Comput. Assist. Radiol. Surg..

[3] Gupta B., Agarwal T.K. (2017). New contrast enhancement approach for dark images with non-uniform illumination. Comput. Electr. Eng..

[4] Jebadurai J., Peter J.D. (2017). SK-SVR: Sigmoid kernel support vector regression based in-scale single image super-resolution. Pattern Recognit. Lett..

[5] Lee C.C., Shih C.Y. Classification of Liver Disease from CT Images Using Sigmoid Radial Basis Function Neural Network. Proceedings of the 2009 WRI World Congress on Computer Science and Information Engineering.

[6] Kondo T., Ueno J., Takao S. (2013). Hybrid Multi-layered GMDH-type Neural Network Using Principal Component Regression Analysis and its Application to Medical Image Diagnosis of Liver Cancer. Procedia Comput. Sci..

[7] Gandhamal A., Talbar S., Gajre S., Hani A.F.M., Kumar D. (2017). Local gray level S-curve transformation: A generalized contrast enhancement technique for medical images. Comput. Biol. Med..

[8] Huang S.M., Liu H.L., Li M.L. Improved temperature imaging of focused ultrasound thermal therapy using a sigmoid model based cross-correlation algorithm. Proceedings of the 2012 IEEE International Ultrasonics Symposium.

[9] Shao H., Zhang Y., Xian M., Cheng H., Xu F., Ding J. A saliency model for automated tumor detection in breast ultrasound images. Proceedings of the 2015 IEEE International Conference on Image Processing—ICIP.

[10] Almajalid R., Shan J., Du Y., Zhang M. Development of a Deep-Learning-Based Method for Breast Ultrasound Image Segmentation. Proceedings of the 2018 17th IEEE International Conference on Machine Learning and Applications (ICMLA).

[11] Luo X., Zhang Z., Zhang C., Wu X. (2017). Multi-focus image fusion using HOSVD and edge intensity. J. Vis. Commun. Image Represent..

[12] Zhang X., Li X., Feng Y. (2016). A new multifocus image fusion based on spectrum comparison. Signal Process..

[13] Luo X., Zhang Z., Wu X. Image Fusion Using Region Segmentation and Sigmoid Function. Proceedings of the 2014 22nd International Conference on Pattern Recognition.

[14] Jung C., Cao L., Liu H., Kim J. (2015). Visual comfort enhancement in stereoscopic 3D images using saliency-adaptive nonlinear disparity mapping. Displays.

[15] Tsallis C., Mendes R., Plastino A. (1998). The role of constraints within generalized nonextensive statistics. Phys. A Stat. Mech. Its Appl..

[16] Tsallis C. (1999). Nonextensive Statistics: Theoretical, Experimental and Computational Evidences and Connections. Braz. J. Phys..

[17] Borges E.P. (1999). Irreversibilidade, Desordem e Incerteza: Tres Visoes da Generalizacao do Conceito de Entropia. Rev. Bras. De Ensino De Fis..

[18] Anastasiadis A. (2012). Special Issue: Tsallis Entropy. Entropy.

[19] Zang W., Wang Z., Jiang D., Liu X., Jiang Z. (2018). Classification of MRI Brain Images Using DNA Genetic Algorithms Optimized Tsallis Entropy and Support Vector Machine. Entropy.

[20] Albuquerque M.P., Albuquerque M.P., Esquef I.A., Mello A.R.G. (2004). Image thresholding using Tsallis entropy. Pattern Recognit. Lett..

[21] Rodrigues P.S., Wachs-Lopes G.A., Erdmann H.R., Ribeiro M.P., Giraldi G.A. (2015). Improving a firefly meta-heuristic for multilevel image segmentation using Tsallis entropy. Pattern Anal. Appl..

[22] Rodrigues P.S., Chang R.F., Giraldi G.A., Suri J.S. Non-extensive entropy for cad systems of breast cancer images. Proceedings of the 19th Brazilian Symposium on Computer Graphics and Image Processing.

[23] Sparavigna A.C. (2015). On the Role of Tsallis Entropy in Image Processing. Int. Sci. Res. J..

[24] Sezgin M., Sankur B. (2004). Survey over image thresholding techniques and quantitative performance evaluation. J. Electron. Imaging.

[25] Ronneberger O., Fischer P., Brox T. (2015). U-Net: Convolutional Networks for Biomedical Image Segmentation. Medical Image Computing and Computer-Assisted Intervention (MICCAI).

[26] Tsallis C. (1994). What are the numbers that experiments provide. Quim. Nova.

[27] Rodrigues P.S., Giraldi G.A., Provenzano M., Faria M.D., Chang R.F., Suri J.S. A new methodology based on q-entropy for breast lesion classification in 3-D ultrasound images. Proceedings of the 28th International Conference of the IEEE Engineering in Medicine and Biology Society—EMBS’06.

[28] Rodrigues P.S., Giraldi G.A. Computing the q-index for Tsallis Nonextensive Image Segmentation. Proceedings of the SIBIGRAPI 2009.

[29] Rodrigues P.S., Giraldi G.A. (2011). Improving the non-extensive medical image segmentation based on Tsallis entropy. Pattern Anal. Appl..

[30] Gonzalez R., Woods R. (2010). Processamento Digital de Imagens.

[31] Serra J. (1982). Image Analysis and Mathematical Morphology.

[32] Chen Y. (2014). An allometric scaling relation based on logistic growth of cities. Chaos Solitons Fractals.

[33] Chen Y.G. (2018). Logistic Models of Fractal Dimension Growth of Urban Morphology. Fractals.

[34] Jain A.K. (1989). Fundamentals of Digital Image Processing.

[35] Giraldi G.A., Rodrigues P.S.S. (2016). Theoretical Elements in Fourier Analysis of q-Gaussian Functions. Theor. Appl. Inform..

[36] Borges E.P., Tsallis C., Miranda J.G.V., Andrade R.F.S. (2004). Mother wavelet functions generalized through q-exponentials. J. Phys. A Math. Gen..

[37] Johal R.S. (1998). q calculus and entropy in nonextensive statistical physics. Phys. Rev. E.

[38] Wachs-Lopes G., Horvath M., Giraldi G., Rodrigues P. (2019). A strategy based on non-extensive statistics to improve frame-matching algorithms under large viewpoint changes. Signal Process. Image Commun..

